# 2-Meth­oxy­anilinium 3-hy­droxy-2,4,6-trinitro­phenolate

**DOI:** 10.1107/S1600536811005708

**Published:** 2011-02-23

**Authors:** Doraisamyraja Kalaivani, Rangasamy Malarvizhi, Kaliyaperumal Thanigaimani, Packianathan Thomas Muthiah

**Affiliations:** a PG & Research Department of Chemistry, Seethalakshmi Ramaswami College, Tiruchirappalli 620 002, Tamil Nadu, India; b Department of Chemistry, Thanthai Hans Roever College, Perambalur 621 212, Tamilnadu, India; c School of Chemistry, Bharathidasan University, Tiruchirappalli 620 024, Tamilnadu, India

## Abstract

The cation and anion of the title mol­ecular salt, C_7_H_10_NO^+^·C_6_H_2_N_3_O_8_^−^, are linked *via* an N—H⋯O hydrogen bond. An intra­molecular O—H⋯O hydrogen bond is also found in the anion. In the crystal, the anions self-assemble *via* O—H⋯O hydrogen bonds, forming a *C*(9) supra­molecular chain the *b* axis. Further inter­molecular N—H⋯O inter­actions also occur.

## Related literature

For crystalline metal complexes of styphnic acid, see: Cui *et al.* (2008**a* ▶,b* ▶); Orbovic & Codoceo (2008 ▶); Zheng *et al.* (2006*a* ▶,*b* ▶); Zhu & Xiao (2009 ▶). For crystalline adducts of styphnic acid with organic bases, see: Abashev *et al.* (2001 ▶); Liu *et al.* (2008 ▶); Tenishev *et al.* (2002 ▶); For related mol­ecular salts, see: Kalaivani & Malarvizhi (2010 ▶); Vogel (1978 ▶). 
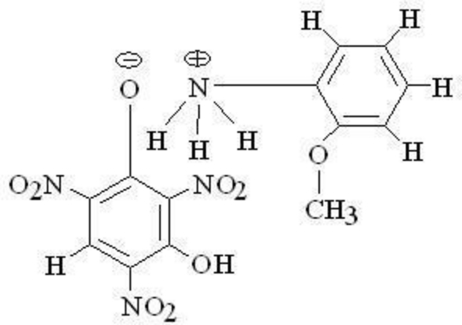


## Experimental

### 

#### Crystal data

C_7_H_10_NO^+^·C_6_H_2_N_3_O_8_^−^
*M* *_r_* = 368.27
Monoclinic, 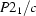

*a* = 10.6957 (4) Å
*b* = 17.8368 (5) Å
*c* = 8.0527 (3) Å
β = 91.991 (2)°
*V* = 1535.34 (9) Å^3^
*Z* = 4
Mo *K*α radiation
μ = 0.14 mm^−1^
*T* = 293 K
0.22 × 0.18 × 0.12 mm

#### Data collection

Bruker SMART APEXII CCD area-detector diffractometer
Absorption correction: multi-scan (*SADABS*; Bruker, 2008 ▶) *T*_min_ = 0.978, *T*_max_ = 0.988
19208 measured reflections
4930 independent reflections
3539 reflections with *I* > 2σ(*I*)
*R*_int_ = 0.028

#### Refinement

*R*[*F* ^2^ > 2σ(*F* ^2^)] = 0.050
*wR*(*F* ^2^) = 0.142
*S* = 1.04
4930 reflections
238 parameters
H-atom parameters constrained
Δρ_max_ = 0.45 e Å^−3^
Δρ_min_ = −0.40 e Å^−3^

### 

Data collection: *APEX2* (Bruker, 2008 ▶); cell refinement: *SAINT* (Bruker, 2008 ▶); data reduction: *SAINT*; program(s) used to solve structure: *SHELXS97* (Sheldrick, 2008 ▶); program(s) used to refine structure: *SHELXL97* (Sheldrick, 2008 ▶); molecular graphics: *PLATON* (Spek, 2009 ▶); software used to prepare material for publication: *PLATON*.

## Supplementary Material

Crystal structure: contains datablocks global, I. DOI: 10.1107/S1600536811005708/bv2174sup1.cif

Structure factors: contains datablocks I. DOI: 10.1107/S1600536811005708/bv2174Isup2.hkl

Additional supplementary materials:  crystallographic information; 3D view; checkCIF report

## Figures and Tables

**Table 1:** Hydrogen-bond geometry (Å, °)

*D*—H⋯*A*	*D*—H	H⋯*A*	*D*⋯*A*	*D*—H⋯*A*
N1—H1*A*⋯O2	0.89	1.89	2.7408 (16)	158
N1—H1*C*⋯O2^i^	0.89	1.87	2.7569 (16)	177
O5—H5⋯O6	0.82	1.95	2.6311 (18)	140
O5—H5⋯O9^ii^	0.82	2.29	2.9263 (17)	135

Symmetry codes: (i) 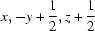; (ii) 
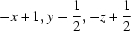
.

**Fig. 1.**
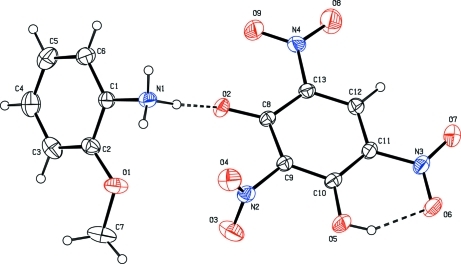
The asymmetric unit of (I), showing 50% probability displacement ellipsoids. Dashed lines indicate hydrogen bonds.

**Fig. 2.**
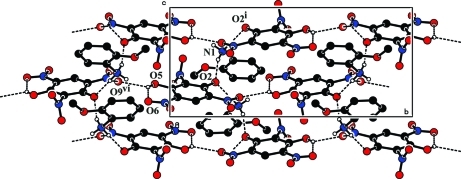
The packing view of 2-methoxyanilinium 3-hydroxy- 2,4,6-trinitrophenolate.Dashed lines indicate hydrogen bonds H atoms not involved in hydrogen bonding have been omitted [symmetry codes: (i) x, -y + 1/2, z + 1/2; (vi) -x + 1, y - 1/2, -z + 1/2].

**Fig. 3.**
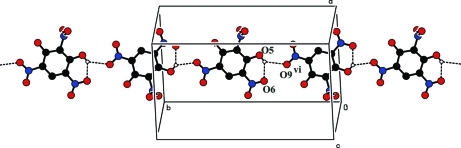
Hydrogen-bonding patterns in the supramolecular chain in compound (I). Dashed lines indicate hydrogen bonds H atoms not involved in hydrogen bonding have been omitted [symmetry codes: (vi) -x + 1, y - 1/2, -z + 1/2].

## supplementary crystallographic information

### Comment

In spite of the fact that many crystalline complexes have been derived from styphnic acid and metals in recent years [(Cui *et al.*, 2008**a*,* 2008*b*), (Orbovic *et al.*, 2008), (Zheng *et al.*, 2006*a*, 2006*b*), (Zhu & Xiao, 2009)], only a few crystalline complexes are known from styphnic acid and organic molecules [(Abashev *et al.*, 2001), (Liu *et al.*, 2008), (Tenishev *et al.*, 2002)]. It has also been pointed out that aromatic hydrocarbons (and also some amines) form 1:1 adducts with styphnic acid and these derivatives do not crystallize as well as the corresponding picrates (Vogel, 1978). In the present work an elegant method has been proposed to prepare a crystalline molecular salt from 2-methoxyaniline and styphnic acid.

The title compound (I), is shown in Fig 1. The adduct formation between styphnic acid and 2-methoxyaniline may involve two important types of charge-transfer interactions (i) π-π^*^ transition and (ii) proton transfer. A view of the crystal packing is shown in Fig 2. The proton transfer from phenolic OH to amino group is the main contributing factor which stabilizes the title molecular salt. The same observation has been reported by us in a related molecular salt (Kalaivani & Malarvizhi, 2010). The 3-hydroxy-2,4,6-trinitrophenolate ions self-assemble *via* O—H···O hydrogen bonds to from a supramolecular chain the *b* axis, with the graph-set notation C(9); this is shown in Fig. 3.

### Experimental

2,4,6-Trinitro-1,3-benzenediol (styphnic acid: 2.45 g, 0.01 mol) was dissolved in the minimum quantity of dimethyl sulphoxide. 2-Methoxyaniline (1.23 g, 0.01 mol) dissolved in the minimum amount of dimethyl sulphoxide was added to styphnic acid solution. The mixture was stirred well for 3 h and kept as such for another 12 h. The mixture was then poured into ice cold water with stirring. The molecular salt (adduct) formed was filtered and washed first with water and then with alcohol and dried. The dried adduct was washed several times with ether and recrystallized from ethanol (yield 70–75%, mp.455 K). Good pale yellow crystals of the molecular salt were obtained by slow evaporation of ethanol at room temperature. The same molecular salt was obtained when styphnic acid (0.01 mol) was mixed with excess of 2-methoxyaniline (0.03 mol).

### Refinement

All hydrogen atoms were positioned geometrically and were refined using a riding model. The C—H, O—H and N—H bond lengths are 0.93–0.96, 0.82 and 0.89 Å, respectively [*U*_iso_ (H)=1.2 *U*_eq_(parent atom)].

### Crystal data

**Table:**

C_7_H_10_NO^+^·C_6_H_2_N_3_O_8_^−^	*F*(000) = 760
*M**_r_* = 368.27	*D*_x_ = 1.593 Mg m^−^^3^
Monoclinic, *P*2_1_/*c*	Mo *K*α radiation, λ = 0.71073 Å
Hall symbol: -P 2ybc	Cell parameters from 4930 reflections
*a* = 10.6957 (4) Å	θ = 1.9–31.8°
*b* = 17.8368 (5) Å	µ = 0.14 mm^−^^1^
*c* = 8.0527 (3) Å	*T* = 293 K
β = 91.991 (2)°	Prism, colourless
*V* = 1535.34 (9) Å^3^	0.22 × 0.18 × 0.12 mm
*Z* = 4	

### Data collection

**Table:**

Bruker SMART APEXII CCD area-detector diffractometer	4930 independent reflections
Radiation source: fine-focus sealed tube	3539 reflections with *I* > 2σ(*I*)
graphite	*R*_int_ = 0.028
φ and ω scans	θ_max_ = 31.8°, θ_min_ = 1.9°
Absorption correction: multi-scan (*SADABS*; Bruker, 2008)	*h* = −15→15
*T*_min_ = 0.978, *T*_max_ = 0.988	*k* = −25→26
19208 measured reflections	*l* = −11→11

### Refinement

**Table:**

Refinement on *F*^2^	Primary atom site location: structure-invariant direct methods
Least-squares matrix: full	Secondary atom site location: difference Fourier map
*R*[*F*^2^ > 2σ(*F*^2^)] = 0.050	Hydrogen site location: inferred from neighbouring sites
*wR*(*F*^2^) = 0.142	H-atom parameters constrained
*S* = 1.04	*w* = 1/[σ^2^(*F*_o_^2^) + (0.0626*P*)^2^ + 0.4547*P*] where *P* = (*F*_o_^2^ + 2*F*_c_^2^)/3
4930 reflections	(Δ/σ)_max_ < 0.001
238 parameters	Δρ_max_ = 0.45 e Å^−^^3^
0 restraints	Δρ_min_ = −0.40 e Å^−^^3^

### Special details

**Table:**

Geometry. Bond distances, angles *etc*. have been calculated using the rounded fractional coordinates. All su's are estimated from the variances of the (full) variance-covariance matrix. The cell e.s.d.'s are taken into account in the estimation of distances, angles and torsion angles
Refinement. Refinement on *F*^2^ for ALL reflections except those flagged by the user for potential systematic errors. Weighted *R*-factors *wR* and all goodnesses of fit *S* are based on *F*^2^, conventional *R*-factors *R* are based on *F*, with *F* set to zero for negative *F*^2^. The observed criterion of *F*^2^ > σ(*F*^2^) is used only for calculating -*R*-factor-obs *etc*. and is not relevant to the choice of reflections for refinement. *R*-factors based on *F*^2^ are statistically about twice as large as those based on *F*, and *R*-factors based on ALL data will be even larger.

### Fractional atomic coordinates and isotropic or equivalent isotropic displacement parameters (Å^2^)

**Table:**

	*x*	*y*	*z*	*U*_iso_*/*U*_eq_	
O1	−0.00430 (12)	0.14533 (7)	0.45345 (16)	0.0536 (4)	
N1	0.18296 (12)	0.21738 (7)	0.59909 (15)	0.0366 (3)	
C1	0.08388 (13)	0.26071 (8)	0.51522 (17)	0.0344 (4)	
C2	−0.01386 (14)	0.22085 (9)	0.43872 (19)	0.0400 (4)	
C3	−0.10912 (16)	0.26008 (12)	0.3561 (2)	0.0529 (6)	
C4	−0.10434 (19)	0.33740 (12)	0.3498 (3)	0.0592 (6)	
C5	−0.0059 (2)	0.37645 (11)	0.4229 (3)	0.0580 (6)	
C6	0.08921 (16)	0.33785 (9)	0.5076 (2)	0.0450 (5)	
C7	−0.1013 (2)	0.10034 (13)	0.3781 (3)	0.0712 (8)	
O2	0.30956 (10)	0.18764 (5)	0.31638 (13)	0.0372 (3)	
O3	0.18239 (12)	0.01420 (9)	0.30014 (18)	0.0662 (5)	
O4	0.26170 (13)	0.05325 (8)	0.53302 (15)	0.0569 (4)	
O5	0.43465 (12)	−0.06180 (6)	0.30043 (17)	0.0508 (4)	
O6	0.64034 (12)	−0.08887 (6)	0.14078 (16)	0.0519 (4)	
O7	0.76246 (12)	0.00182 (7)	0.07391 (18)	0.0585 (4)	
O8	0.63270 (16)	0.25143 (8)	0.0819 (3)	0.0929 (7)	
O9	0.46083 (14)	0.28678 (7)	0.1770 (3)	0.0813 (7)	
N2	0.26692 (12)	0.04141 (7)	0.38421 (16)	0.0376 (4)	
N3	0.66485 (12)	−0.02118 (7)	0.12862 (16)	0.0405 (4)	
N4	0.53537 (12)	0.23793 (7)	0.14816 (19)	0.0428 (4)	
C8	0.39391 (12)	0.14238 (7)	0.27345 (16)	0.0299 (3)	
C9	0.38107 (13)	0.06399 (7)	0.30251 (17)	0.0323 (4)	
C10	0.46368 (14)	0.00851 (7)	0.26041 (18)	0.0346 (4)	
C11	0.57235 (13)	0.03198 (8)	0.17977 (18)	0.0346 (4)	
C12	0.59200 (13)	0.10722 (8)	0.14638 (18)	0.0353 (4)	
C13	0.50678 (13)	0.16065 (7)	0.19047 (17)	0.0326 (4)	
H1A	0.23360	0.19870	0.52400	0.0550*	
H1B	0.14930	0.18000	0.65560	0.0550*	
H1C	0.22640	0.24700	0.66890	0.0550*	
H3	−0.17580	0.23450	0.30530	0.0630*	
H4	−0.16870	0.36360	0.29510	0.0710*	
H5A	−0.00330	0.42850	0.41560	0.0700*	
H6	0.15570	0.36370	0.55850	0.0540*	
H7A	−0.17980	0.11290	0.42510	0.1070*	
H7B	−0.08320	0.04830	0.39790	0.1070*	
H7C	−0.10580	0.10960	0.26060	0.1070*	
H5	0.49030	−0.09010	0.27160	0.0760*	
H12	0.66400	0.12170	0.09330	0.0420*	

### Atomic displacement parameters (Å^2^)

**Table:**

	*U*^11^	*U*^22^	*U*^33^	*U*^12^	*U*^13^	*U*^23^
O1	0.0559 (7)	0.0422 (6)	0.0617 (8)	−0.0173 (5)	−0.0108 (6)	−0.0007 (5)
N1	0.0394 (6)	0.0344 (6)	0.0360 (6)	−0.0046 (5)	0.0009 (5)	−0.0036 (5)
C1	0.0349 (7)	0.0371 (7)	0.0316 (6)	−0.0025 (5)	0.0071 (5)	−0.0028 (5)
C2	0.0382 (7)	0.0448 (8)	0.0372 (7)	−0.0080 (6)	0.0054 (6)	−0.0011 (6)
C3	0.0390 (8)	0.0708 (12)	0.0487 (9)	−0.0041 (8)	−0.0011 (7)	0.0013 (8)
C4	0.0530 (10)	0.0678 (12)	0.0567 (11)	0.0164 (9)	0.0010 (9)	0.0078 (9)
C5	0.0692 (12)	0.0435 (9)	0.0616 (11)	0.0104 (8)	0.0064 (10)	0.0029 (8)
C6	0.0491 (9)	0.0380 (8)	0.0484 (9)	−0.0038 (6)	0.0070 (7)	−0.0043 (6)
C7	0.0722 (13)	0.0657 (12)	0.0750 (14)	−0.0377 (11)	−0.0093 (11)	−0.0021 (10)
O2	0.0427 (5)	0.0302 (5)	0.0391 (5)	0.0093 (4)	0.0059 (4)	0.0036 (4)
O3	0.0518 (7)	0.0849 (10)	0.0619 (9)	−0.0254 (7)	0.0013 (6)	−0.0089 (7)
O4	0.0685 (8)	0.0630 (8)	0.0397 (6)	−0.0072 (6)	0.0112 (6)	0.0038 (5)
O5	0.0551 (7)	0.0250 (5)	0.0729 (8)	0.0048 (5)	0.0093 (6)	0.0032 (5)
O6	0.0591 (7)	0.0340 (6)	0.0626 (8)	0.0103 (5)	0.0011 (6)	−0.0118 (5)
O7	0.0470 (7)	0.0551 (7)	0.0744 (9)	0.0086 (6)	0.0155 (6)	−0.0091 (6)
O8	0.0737 (10)	0.0508 (8)	0.1580 (18)	−0.0022 (7)	0.0568 (11)	0.0234 (10)
O9	0.0604 (8)	0.0281 (6)	0.1575 (17)	0.0013 (6)	0.0329 (10)	0.0079 (8)
N2	0.0429 (7)	0.0294 (5)	0.0407 (7)	−0.0008 (5)	0.0036 (5)	0.0046 (5)
N3	0.0436 (7)	0.0376 (6)	0.0400 (7)	0.0088 (5)	−0.0023 (5)	−0.0090 (5)
N4	0.0378 (6)	0.0310 (6)	0.0595 (8)	−0.0029 (5)	0.0004 (6)	0.0055 (5)
C8	0.0339 (6)	0.0269 (6)	0.0286 (6)	0.0017 (5)	−0.0021 (5)	−0.0003 (4)
C9	0.0346 (6)	0.0269 (6)	0.0353 (7)	0.0002 (5)	0.0018 (5)	0.0003 (5)
C10	0.0407 (7)	0.0257 (6)	0.0371 (7)	0.0021 (5)	−0.0037 (6)	−0.0022 (5)
C11	0.0355 (7)	0.0317 (6)	0.0363 (7)	0.0059 (5)	−0.0017 (5)	−0.0052 (5)
C12	0.0326 (6)	0.0359 (7)	0.0373 (7)	0.0010 (5)	−0.0003 (5)	−0.0026 (5)
C13	0.0338 (6)	0.0262 (6)	0.0375 (7)	−0.0012 (5)	−0.0020 (5)	0.0002 (5)

### Geometric parameters (Å, °)

**Table:**

O1—C2	1.356 (2)	C1—C6	1.379 (2)
O1—C7	1.430 (3)	C2—C3	1.387 (2)
O2—C8	1.2676 (16)	C3—C4	1.381 (3)
O3—N2	1.2119 (19)	C4—C5	1.377 (3)
O4—N2	1.2200 (18)	C5—C6	1.388 (3)
O5—C10	1.3342 (17)	C3—H3	0.9300
O6—N3	1.2401 (17)	C4—H4	0.9300
O7—N3	1.2183 (18)	C5—H5A	0.9300
O8—N4	1.210 (2)	C6—H6	0.9300
O9—N4	1.2090 (19)	C7—H7C	0.9600
O5—H5	0.8200	C7—H7A	0.9600
N1—C1	1.4583 (19)	C7—H7B	0.9600
N1—H1C	0.8900	C8—C13	1.4374 (19)
N1—H1A	0.8900	C8—C9	1.4251 (18)
N1—H1B	0.8900	C9—C10	1.3769 (19)
N2—C9	1.4634 (19)	C10—C11	1.414 (2)
N3—C11	1.4408 (19)	C11—C12	1.386 (2)
N4—C13	1.4550 (18)	C12—C13	1.3739 (19)
C1—C2	1.390 (2)	C12—H12	0.9300
			
O1···N1	2.6221 (18)	N3···C10^iii^	3.3846 (19)
O1···C4^i^	3.415 (3)	N4···O2	2.9496 (17)
O2···C1	3.2183 (17)	N3···H5	2.5400
O2···N1^ii^	2.7569 (16)	C1···O2	3.2183 (17)
O2···C6^ii^	3.397 (2)	C1···C3^i^	3.511 (2)
O2···O4	3.0189 (17)	C2···C3^i^	3.562 (2)
O2···O9	2.6703 (19)	C3···C2^ii^	3.562 (2)
O2···N1	2.7408 (16)	C3···O8^xi^	3.365 (3)
O2···N2	2.7068 (15)	C3···C1^ii^	3.511 (2)
O2···N4	2.9496 (17)	C4···O1^ii^	3.415 (3)
O3···O5	3.0194 (18)	C6···O7^ix^	3.402 (2)
O3···O7^iii^	3.103 (2)	C6···O2^i^	3.397 (2)
O4···N1	3.0974 (19)	C7···O7^xii^	3.311 (3)
O4···O2	3.0189 (17)	C7···O4^v^	3.324 (3)
O4···C11^iv^	3.2443 (19)	C10···N3^iii^	3.3846 (19)
O4···C7^v^	3.324 (3)	C11···C11^iii^	3.431 (2)
O4···N3^iv^	2.8670 (18)	C11···O4^iv^	3.2443 (19)
O4···O6^iv^	2.8654 (18)	C12···O6^iii^	3.3512 (19)
O5···N3	2.9560 (18)	C13···O6^iii^	3.3074 (19)
O5···O6	2.6311 (18)	C3···H7C	2.7900
O5···O9^vi^	2.9263 (17)	C3···H7A	2.7900
O5···N2	2.6733 (17)	C5···H7C^i^	2.9700
O5···O3	3.0194 (18)	C5···H1B^ii^	2.9400
O6···O9^vi^	2.890 (2)	C6···H1B^ii^	2.9500
O6···C13^iii^	3.3074 (19)	C7···H3	2.5800
O6···C12^iii^	3.3512 (19)	C8···H6^ii^	3.0300
O6···O5	2.6311 (18)	C8···H1A	2.8700
O6···O4^iv^	2.8654 (18)	C8···H1C^ii^	2.7800
O7···C6^vi^	3.402 (2)	H1A···O1	2.7600
O7···O3^iii^	3.103 (2)	H1A···O9^i^	2.7000
O7···C7^vii^	3.311 (3)	H1A···O2	1.8900
O8···C3^viii^	3.365 (3)	H1A···O4	2.6100
O9···O6^ix^	2.890 (2)	H1A···C8	2.8700
O9···O5^ix^	2.9263 (17)	H1B···C5^i^	2.9400
O9···N1^ii^	3.017 (2)	H1B···C6^i^	2.9500
O9···O2	2.6703 (19)	H1B···O4	2.7600
O1···H1A	2.7600	H1B···O1	2.3500
O1···H1B	2.3500	H1C···H6	2.3800
O2···H1C^ii^	1.8700	H1C···O9^i^	2.5800
O2···H1A	1.8900	H1C···C8^i^	2.7800
O2···H6^ii^	2.7600	H1C···O2^i^	1.8700
O3···H7B^v^	2.9100	H3···H7A	2.3700
O3···H4^x^	2.8000	H3···H7C	2.3800
O4···H1B	2.7600	H3···O8^xii^	2.7000
O4···H7B^v^	2.7000	H3···C7	2.5800
O4···H1A	2.6100	H4···O3^xiii^	2.8000
O6···H6^vi^	2.8800	H5···N3	2.5400
O6···H5	1.9500	H5···O9^vi^	2.2900
O7···H7C^vii^	2.7900	H5···O6	1.9500
O7···H12	2.3900	H5A···O7^ix^	2.8900
O7···H6^vi^	2.8400	H6···H1C	2.3800
O7···H5A^vi^	2.8900	H6···O6^ix^	2.8800
O8···H3^vii^	2.7000	H6···O7^ix^	2.8400
O8···H12	2.3400	H6···O2^i^	2.7600
O9···H1A^ii^	2.7000	H6···C8^i^	3.0300
O9···H5^ix^	2.2900	H7A···H3	2.3700
O9···H1C^ii^	2.5800	H7A···C3	2.7900
N1···O9^i^	3.017 (2)	H7B···O4^v^	2.7000
N1···O2	2.7408 (16)	H7B···O3^v^	2.9100
N1···O1	2.6221 (18)	H7C···O7^xii^	2.7900
N1···O4	3.0974 (19)	H7C···H3	2.3800
N1···O2^i^	2.7569 (16)	H7C···C5^ii^	2.9700
N2···O5	2.6733 (17)	H7C···C3	2.7900
N2···O2	2.7068 (15)	H12···O7	2.3900
N3···O4^iv^	2.8670 (18)	H12···O8	2.3400
N3···O5	2.9560 (18)		
			
C2—O1—C7	117.97 (14)	C5—C4—H4	119.00
C10—O5—H5	109.00	C6—C5—H5A	120.00
H1B—N1—H1C	109.00	C4—C5—H5A	120.00
C1—N1—H1B	109.00	C5—C6—H6	120.00
C1—N1—H1A	109.00	C1—C6—H6	120.00
H1A—N1—H1C	110.00	O1—C7—H7C	109.00
C1—N1—H1C	109.00	O1—C7—H7B	109.00
H1A—N1—H1B	109.00	H7B—C7—H7C	109.00
O4—N2—C9	117.47 (13)	H7A—C7—H7B	110.00
O3—N2—C9	118.49 (13)	H7A—C7—H7C	110.00
O3—N2—O4	124.01 (14)	O1—C7—H7A	109.00
O6—N3—C11	117.97 (12)	O2—C8—C9	120.43 (12)
O7—N3—C11	119.17 (12)	C9—C8—C13	112.71 (11)
O6—N3—O7	122.86 (13)	O2—C8—C13	126.84 (12)
O8—N4—O9	121.62 (15)	N2—C9—C10	117.74 (12)
O9—N4—C13	119.50 (14)	C8—C9—C10	126.76 (13)
O8—N4—C13	118.86 (13)	N2—C9—C8	115.50 (11)
C2—C1—C6	121.49 (14)	O5—C10—C11	126.23 (13)
N1—C1—C6	121.27 (13)	C9—C10—C11	116.42 (12)
N1—C1—C2	117.21 (13)	O5—C10—C9	117.35 (13)
C1—C2—C3	118.89 (15)	N3—C11—C12	118.13 (12)
O1—C2—C1	114.58 (13)	C10—C11—C12	120.54 (13)
O1—C2—C3	126.53 (15)	N3—C11—C10	121.33 (12)
C2—C3—C4	119.60 (17)	C11—C12—C13	121.00 (13)
C3—C4—C5	121.20 (19)	N4—C13—C12	116.74 (12)
C4—C5—C6	119.71 (18)	C8—C13—C12	122.57 (12)
C1—C6—C5	119.10 (16)	N4—C13—C8	120.69 (12)
C4—C3—H3	120.00	C11—C12—H12	119.00
C2—C3—H3	120.00	C13—C12—H12	120.00
C3—C4—H4	119.00		
			
C7—O1—C2—C1	179.98 (15)	C3—C4—C5—C6	−1.4 (3)
C7—O1—C2—C3	−0.4 (2)	C4—C5—C6—C1	0.8 (3)
O4—N2—C9—C10	105.00 (16)	O2—C8—C9—C10	178.89 (14)
O3—N2—C9—C8	102.38 (16)	C13—C8—C9—C10	0.3 (2)
O4—N2—C9—C8	−75.60 (17)	O2—C8—C13—N4	0.7 (2)
O3—N2—C9—C10	−77.02 (18)	C9—C8—C13—N4	179.16 (13)
O7—N3—C11—C10	−173.12 (14)	C13—C8—C9—N2	−179.01 (12)
O6—N3—C11—C12	−171.69 (14)	O2—C8—C13—C12	−178.89 (14)
O7—N3—C11—C12	7.5 (2)	C9—C8—C13—C12	−0.44 (19)
O6—N3—C11—C10	7.7 (2)	O2—C8—C9—N2	−0.45 (19)
O8—N4—C13—C8	178.25 (17)	N2—C9—C10—O5	−1.3 (2)
O9—N4—C13—C8	−3.1 (2)	C8—C9—C10—C11	−0.1 (2)
O8—N4—C13—C12	−2.1 (2)	N2—C9—C10—C11	179.24 (12)
O9—N4—C13—C12	176.54 (18)	C8—C9—C10—O5	179.33 (14)
N1—C1—C6—C5	178.46 (16)	C9—C10—C11—N3	−179.46 (13)
C2—C1—C6—C5	0.6 (2)	C9—C10—C11—C12	−0.1 (2)
N1—C1—C2—C3	−179.29 (13)	O5—C10—C11—C12	−179.44 (15)
C6—C1—C2—O1	178.32 (14)	O5—C10—C11—N3	1.2 (2)
N1—C1—C2—O1	0.41 (19)	C10—C11—C12—C13	0.0 (2)
C6—C1—C2—C3	−1.4 (2)	N3—C11—C12—C13	179.36 (13)
C1—C2—C3—C4	0.8 (2)	C11—C12—C13—N4	−179.30 (13)
O1—C2—C3—C4	−178.90 (17)	C11—C12—C13—C8	0.3 (2)
C2—C3—C4—C5	0.6 (3)		

Symmetry codes: (i) *x*, −*y*+1/2, *z*+1/2; (ii) *x*, −*y*+1/2, *z*−1/2; (iii) −*x*+1, −*y*, −*z*; (iv) −*x*+1, −*y*, −*z*+1; (v) −*x*, −*y*, −*z*+1; (vi) −*x*+1, *y*−1/2, −*z*+1/2; (vii) *x*+1, *y*, *z*; (viii) *x*+1, −*y*+1/2, *z*−1/2; (ix) −*x*+1, *y*+1/2, −*z*+1/2; (x) −*x*, *y*−1/2, −*z*+1/2; (xi) *x*−1, −*y*+1/2, *z*+1/2; (xii) *x*−1, *y*, *z*; (xiii) −*x*, *y*+1/2, −*z*+1/2.

### Hydrogen-bond geometry (Å, °)

**Table:**

*D*—H···*A*	*D*—H	H···*A*	*D*···*A*	*D*—H···*A*
N1—H1A···O2	0.89	1.89	2.7408 (16)	158
N1—H1C···O2^i^	0.89	1.87	2.7569 (16)	177
N1—H1C···O9^i^	0.89	2.58	3.017 (2)	111
O5—H5···O6	0.82	1.95	2.6311 (18)	140
O5—H5···N3	0.82	2.54	2.9560 (18)	112
O5—H5···O9^vi^	0.82	2.29	2.9263 (17)	135
C12—H12···O8	0.93	2.34	2.663 (2)	100

Symmetry codes: (i) *x*, −*y*+1/2, *z*+1/2; (vi) −*x*+1, *y*−1/2, −*z*+1/2.
